# A LSTM-Hawkes hybrid model for posterior click distribution forecast in the advertising network environment

**DOI:** 10.1371/journal.pone.0232887

**Published:** 2020-06-05

**Authors:** Sangwon Hwang, Inwhee Joe

**Affiliations:** Department of Computer Science and Engineering, Hanyang University, Seoul, South Korea; Charles Sturt University, AUSTRALIA

## Abstract

In the field of advertising technology, it is a key task to forecast posterior click distribution since 66% of advertising transactions depend on cost per click model. However, due to the General Data Protection Regulation, machine learning techniques to forecast posterior click distribution based on the sequences of an identified user’s actions are restricted in European countries. To overcome this barrier, we introduce a contextual behavior concept for the advertising network environment and propose a new hybrid model, which we call the Long Short Term Memory—Hawkes model by combining a stochastic-based generative model and a machine learning-based predictive model. Also, to meet the computational efficiency for the heavy demand in mobile advertisement market, we define gradient exponential kernel with just three hyper parameters to minimize residuals. We have carefully tested our proposed model with production data and found that the LSTM-Hawkes model reduces the Mean Squared Error by at least 27.1% and up to 83.8% on average in comparison to the existing Hawkes Process based algorithm, Hawkes Intensity Process, as well as 39.77% on average in comparison to Multivariate Linear Regression. We have also found that our proposed model improves the forecast accuracy by about 21.2% on average.

## Introduction

For the first time in 2017, global digital advertisement expenditure was 41% of the global advertising market, which exceeded the TV advertisement expenditure by 6%. Especially the mobile advertisement in digital marketing recorded 37.6% of the global growth rate, which is an impressive increase, enough to draw attention in the worldwide advertisement market [1]. These reports [2–4] substantiate that the mobile advertising market is leading the growth in the global advertising market. However, despite the rapid market growth, there is a controversial issue that Ad Tech faces. General Data Protection Regulation (GDPR) [5] has been actively enforced by EU since 25 May 2018.

### Related work

The typical strategy of user targeting is based on analyzing the behavior of users across the internet or across the ad network individuals [6, 7, 8]. Another strategy which predicts if someone is going to click an impression is matrix factorization inferring the relationship between user vector and context vector where the output is click through ratio [9, 10, 11]. However, the both traditional methods require user information or inferred user identification as the essential asset to forecast the click popularity or probability. Tracking user behavior between different ad tech individuals has been forbidden in EU as well as personal information transfer is also banned. Thus, technically both types of traditional methods are not feasible to be an real world application under GDPR. To overcome the barrier, we focus on an advertising network’s posterior click distribution; not a user’s CTR, nor an advertisement’s CTR since 1)an advertising network or a publisher is still an identical user group remaining individual users anonymous, and 2)CTR only reflects short-term user engagement. We developed a model that can learn a general and efficient representation of the underlying dynamics from the event history with a hyper-parametric form.

### Advertising network environment

**Fig 1:**
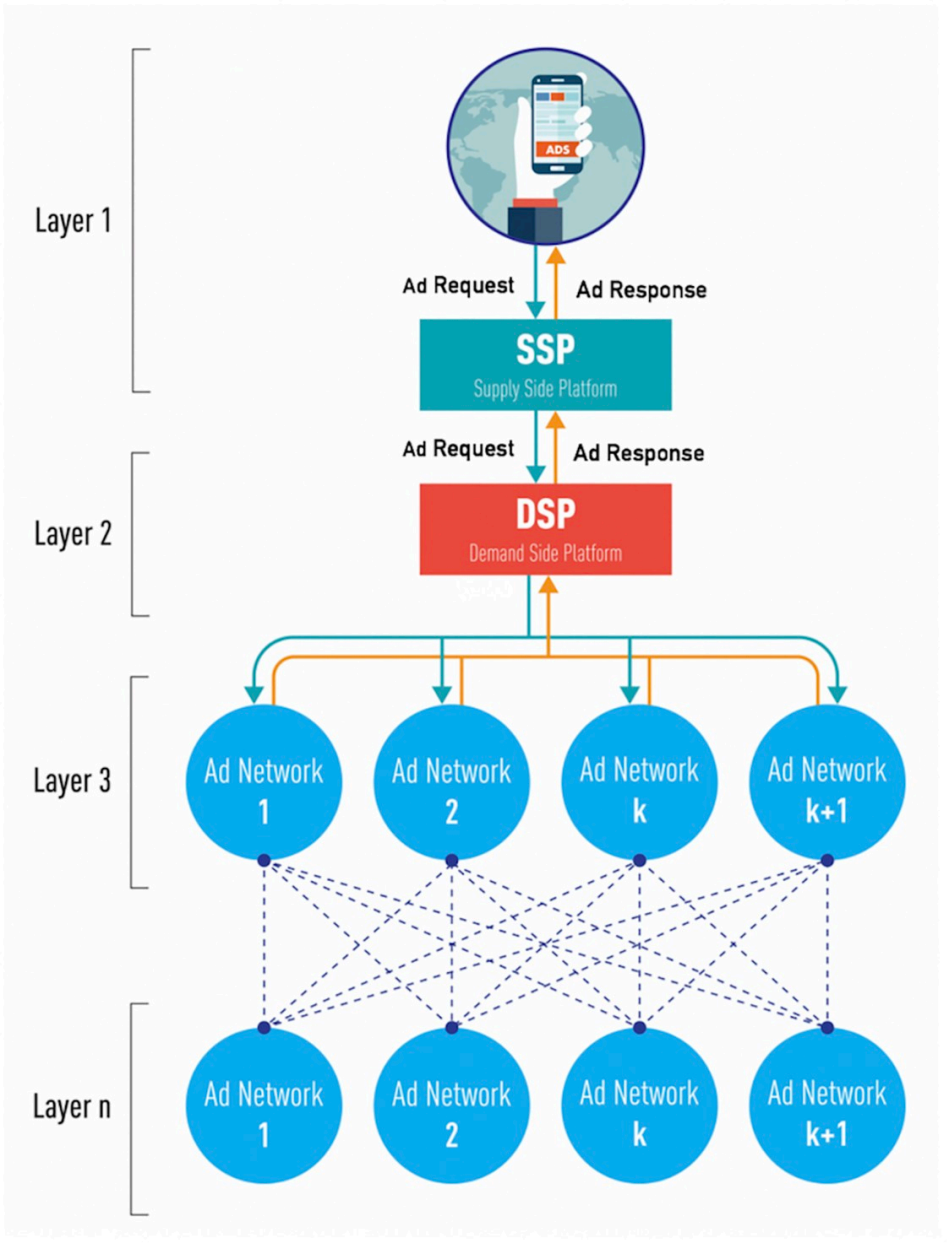
Advertising network architecture. Using Charles, a debugging proxy server application [12], we captured and analyzed packets from an advertising network and found out that the advertising network environment has a multilayer structure with the cycle flow as shown above. Advertising network environment is composed of advertisers (DSP), suppliers (SSP), and advertising networks (Ad Networks).

An advertising network’s environment consists of three main parts. First there is the advertiser who wants to advertise, as well as provide the actual advertisement contents. Next, there is the publisher who provides the landing pages for the advertisement contents. Lastly there is the advertising network that connects the advertiser and publisher together. Advertisers are grouped and managed in a system called the ‘Demand Side Platform’ and publishers are grouped together in a system called the ‘Supply Side Platform’. Fig 1 simply describes the Ad Tech environment and its layers. ***Between layers***, due to the GDPR, user information including advertising IDs, IP addresses or any other unique device IDs from a mobile device cannot be transferred.

## Materials and methods

### Formulation and Preliminary Theory

When a random variable following a Bernoulli process which is the time of occurrence (or success) in Bernoulli trials approaches to positive infinity, it is infeasible to calculate the probability of a specific event occurrence due to the computational complexity. Therefore, to solve this problem, in modeling a posterior distribution of either prediction or simulation, most conventional statistical approaches have adopted ‘Binomial Approximation to Poisson’ which helps find probability of an independent trial in various fields [13–15]. However, the ‘Binomial Approximation to Poisson’ has Memoryless Property [16] because each trial is independent (Independent Increment) and the probability does not change (Stationary Increment). Thus, only event process with non-overlapping intervals can be used in the Binomial Approximation to Poisson in modeling a distribution.

In a click event distribution, where the event occurence time is *t*_*i*_ of set T_*j*_ = {*t*_1_, *t*_2_, …, *t*_*n*_}, inter-arrival time *l*_*i*_ of set *L*_*i*_ = {*l*_1_, *l*_2_, …, *l*_*n*_} can be written as follows

$$\begin{array}{c} t_{n-1}\leq l_{n}<t_{n}\;\;\Rightarrow l_{n}=\left[t_{n-1},t_{n}\right). \end{array}$$

We then can easily find the concurrent occurrences in any of real world’s advertising click distribution. To solve the memoryless property and handle the overlapping intervals, we propose a generative model based on a self-exciting process, a type of non-homogenous process, called Hawkes [17]. In this chapter, we provide mathematical induction from binomial distribution to Hawkes process to derive memory kernel of Hawkes.

#### Binomial approximation to poisson

Suppose that a random variable *X* follows binomial distribution *B*(*n*, *p*), and that the expected value of *X* is λ. When *n* is close to positive infinity, λ approximates to *np* and by binomial approximation to the Poisson distribution, the probability of *X* approximates to the probability mass function of a Poisson distribution.

$$\begin{array}{cc} f(x) & =\left(n!/x!\left(n-x\right)!\right){\left(\lambda /n\right)}^{x}{\left(\left(1-\lambda \right)/n\right)}^{n-x},\\ \lim_{n\to \infty }f\left(x\right) & =\left({\text{exp}}^{\lambda }\lambda^{x}\right)/x!\;where\;\;X∼B\left(n,p\right). \end{array}$$

#### Process

When time unit expands or reduces to by *t* (*t* > 0), the average event occurrences becomes λ*t* during the updated time unit where the average occurrences per default time unit is λ = *np*. Distribution with expanded (or reduced) time unit by *t* is called Process. And, correspondingly, PDF of the process becomes

$$\begin{array}{c} f\left(x,\lambda t\right)=({\text{exp}}^{-\lambda t}{\left(\lambda t\right)}^{x})/x!. \end{array}$$ (1)

#### Poisson distribution and exponential distribution

Suppose that time period for a specific event to take place is a probability variable *t* and that *T* is the time when a specific event *X*_*T*_ takes place. Under this circumstance, the probability of that event *X*_*T*_ occurs after *t* is equal to that the event *X*_*T*_ does not take place within *t*. The PDF of Poisson distribution becomes following equation

$$\begin{array}{c} P\left(t<T\right)=f\left(X_{T}=0,\lambda t\right)=\left({\text{exp}}^{-\lambda t}{\left(\lambda t\right)}^{0}\right)/0!={\text{exp}}^{-\lambda t}. \end{array}$$ (2)

Set Eq (2) as *S*(*t*) then, the probability of that event *X*_*T*_ occurs is 1 − *S*(*t*) which is cumulative distribution function (CDF) for the probability variable *t*. Set this function as *F*(*t*) as following Eq (3)

$$\begin{array}{c} F\left(t\right)=P\left(0\leq T\leq t\right)=1-{\text{exp}}^{-\lambda t}. \end{array}$$ (3)

Since derivative of CDF is PDF, PDF for the random variable t is

$$\begin{array}{c} f\left(t\right)=\frac{d}{dt}F\left(t\right)=\lambda {\text{exp}}^{-\lambda t}. \end{array}$$ (4)

Finally, it is concluded that the probability variable *t* follows exponential distribution with *f*(*t*) and *F*(*t*), PDF and CDF respectively.

#### Non-homogeneous process

Lambda intensity function λ(*t*) determines non-homogeneity. If the event dynamics has positive covariance, intensity function becomes non-homogeneous, known as self exciting, otherwise it becomes homogenous.

$$\begin{array}{c} COV(N(s,t),N(t,u))>0 \end{array}$$ (5)

where *N* is point process and distance (*s*, *t*), (*t*, *u*) satisfy *s* < *x* < *t* and *s* < *t* < *u*.

Mathmatically the intensity function of non-homogeneous processes is defined as the instantaneous rate at which events occur [18]. However, in the real world implementation for Hawkes, technically it has to be the conditional probability of a specific event occurrence during Δ*t*, which can be interpreted as time unit, at *t* where the event has not taken place by *t*. Thus, we suppose that limitation of time unit does not go to zero. We specify each event with the index variable *i* and suppose that the set of event times is history *H*_*t*_. Therefor, we can achieve following equations Eq (5) from Eqs (2), (3) and (4).

$$\begin{array}{cc} \lambda^{*}\left(t\right)= & f\left(t|H_{t}\right)/S\left(t|H_{t}\right)\\ = & f\left(t|H_{t}\right)/\left(1-F\left(t|H_{t}\right)\right)\\ = & P(t_{i+1}\in \left[t,t+\Delta t\right],t_{i+1}\notin \left\{\left(t_{i},t\right),H_{t}\right\})/\Delta t\\ = & E(N\left(t+\Delta t\right)-N\left(t\right)|H_{t})/\Delta t \end{array}$$ (6)

where *H*_*t*_ = {*t*_1_, *t*_2_, …, *t*_*i*_} and *N*(*T*), a counting process, is number of event occurrences during *T*.

### LSTM-Hawkes hybrid model

The maximum likelihood estimation using exponential kernel has a problem with residuals because they become multiplied by real numbers depending on the batch size. We solved this problem by scaling cumulative intensity value of the kernel with differential coefficient *γ* that minimizes the residuals, defined Gradient Exponential Kernel Eq (9).

Also, to forecast posterior event time *t*_*i*_, we used LSTM [19] instead of Thinning algorithm [20] which has been dominantly used with Hawkes, for accuracy enhancement.

In this chapter, we first list all variables and parameters used in the proposed model and define the memory kernel that we suggest. Second, in the subsection ‘Sampling Method’, we describe LSTM architecture and show the test result that empirically proves LSTM is more accurate than Thinning algorithm. Lastly, we propose the LSTM-Hawkes Forecast algorithm [Flow Diagram 1] which combines the generative model with LSTM prediction to draw the posterior process of advertising click event.

#### Hawkes process and its memory kernel

Hawkes process is self-exiting which satisfies Eq (5) in which the intensity rate Eq (6) explicitly and proportionally increases depending on past event occurrences. Hawkes process is formed as Eq (7) where *μ*(*t*) is the background intensity based on the observed activities which can be represented in two ways 1) expected intensity at t based on the observed endogenous events (in our case click) or 2) background intensity describing arrivals of events triggered by exogonous event (in our case conversion). Also hyper parameter *α* increases intensity rate by *α* at each *t*_*i*_ then decreases exponentially back toward *μ*(*t*). Both parameters are estimated by maximum likelihood and the initial value of *μ*(0) is set to expected CTR per time unit in this research.

$$\begin{array}{cc} \lambda (t) & =\mu \left(t\right)+\sum_{t_{i}<t}\phi \left(t-t_{i}\right),\\ \lambda (t) & =\mu \left(t\right)+\int_{-\infty }^{t}\phi \left(t-t_{i}\right)dN\left(t_{i}\right). \end{array}$$ (7)

Commonly used memory kernels with Hawkes process are Exponential kernel, original model proposed by Hawkes, and Power Law kernel, frequently used in social network model [21–23], proposed by Ozaki [24]. In the paper, we have chosen the exponential kernel Eq (8) to achieve better-case time complexity since the number of elementary operations performed increase depending on the umber of variables to approximate. The compared algorithm Eq (14) in our experiments adopts power law kernel Eq (9).

$$\begin{array}{c} \phi (t-t_{i})=\alpha e^{-\delta (t-t_{i})}, \end{array}$$ (8)

$$\begin{array}{c} \phi (t-t_{i})=km^{\beta }{\left(t-t_{i}+c\right)}^{-(1+\theta )}. \end{array}$$ (9)

*Gradient exponential kernel* Gradient exponential kernel is defined as Eq (10), and the code implemented with consecutive loops of while, notated in a pseudo code of Algorithm 1, 2, 3, and 4 where the domain for the memory kernel satisfies *t* ∈ *eP* as following

$$\begin{array}{c} \phi (t-t_{i})=\gamma \alpha e^{-\delta (t-t_{i})}\;where\;t\in eP. \end{array}$$ (10)

#### Hyper parameter estimation

Definition of the lambda intensity function λ(*t*) in non-homogeneous processes was presented in the subchapter ‘Formulation and Preliminary Theory’ as Eq (6) as well as we derive our own memory kernel as Eq (10). By minimizing negative log likelihood (NLL) over the observed data, hyper parameters for the memory kernel can be approximated.

*f*(*t*) Eq (4) is PDF of homogeneous Poisson process. Let *f*′(*t*) is conditional probability on associated history up to *t*_*i*−1_ which is $f^{{}^{\prime }}\left(t\right)=\prod_{i=1}^{T}f\left(t_{i}|H_{i-1}\right)$ then Eqs (2) and (3) can also be re-defined by *f*′(*t*) as *S*′(*t*) and *f*′(*t*) respectively. Correspondingly, likelihood function for Hawkes can be derived as Eq (11) and its log likelihood function as Eq (12), driven by Rubin [25, 26]

$$\begin{array}{c} L(t_{i}|\theta )=\prod_{i=1}^{T}f^{{}^{\prime }}\left(t_{i}|\theta \right)=\prod_{i=1}^{T}\lambda \left(t_{i}|\theta \right)\left(1-F^{{}^{\prime }}\left(t_{i}|\theta \right)\right), \end{array}$$ (11)

$$\begin{array}{c} l(t_{i}|\theta )=-\int_{0}^{T}\lambda \left(t_{i}|\theta \right)dt_{i}+\int_{0}^{T}log\lambda \left(t_{i}|\theta \right)dt_{i}. \end{array}$$ (12)

Therefore, when *eP*_*j*_ is the domain elements for the random variable *t* with PDF *f*′(*t*) and the memory kernel is Eq (10), we can derive NLL function for Gradient Exponential Kernel as follows

$$\begin{array}{c} -l(\tau_{1},\ldots ,\tau_{n}|\theta )=\mu \cdot \tau_{n}-\sum_{m=1}^{n}\left(\gamma \alpha /\delta \right)\left(e^{-\delta \cdot \tau_{m}}-1\right)-\sum_{m=1}^{n}log\left(\mu +\gamma \alpha A\left(m\right)\right), \end{array}$$ (13)

where $A\left(m\right)=\sum_{t_{i}<eP_{m}}e^{-\delta (ep_{m}-t_{i})}\;\text{for}\;i\geq 2$, *t*_*i*_ denotes the event time and A(1) is equal to 0. In our proposed model, optimization is proceeded by minimizing the negative log likelihood Eq (12).

#### Contextual behavior

To define behavior model, we introduce the behavior concept from existing models [27, 28]. User group behavior in advertising network environments is defined based on a set of activities. Also, action sequence, a set of endogenous and exogenous evnets, comprises each activity as element. The behavior model describes how specific ad-networks or publishers perform activities and how often the actions occur at different time.

**Fig 2:**
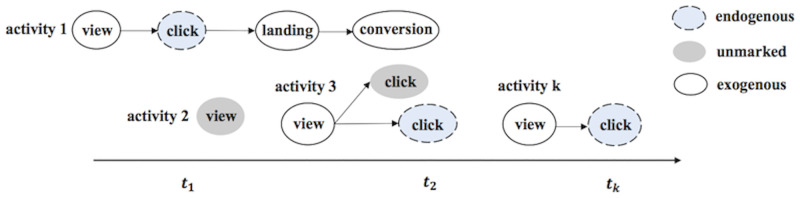
Behavior model in an Ad network. In the case where a user just view an advertisement (no click occurrence), user group’s activity is not temporally marked up since view is not the main interest event in our experiment.

**Fig 3:**
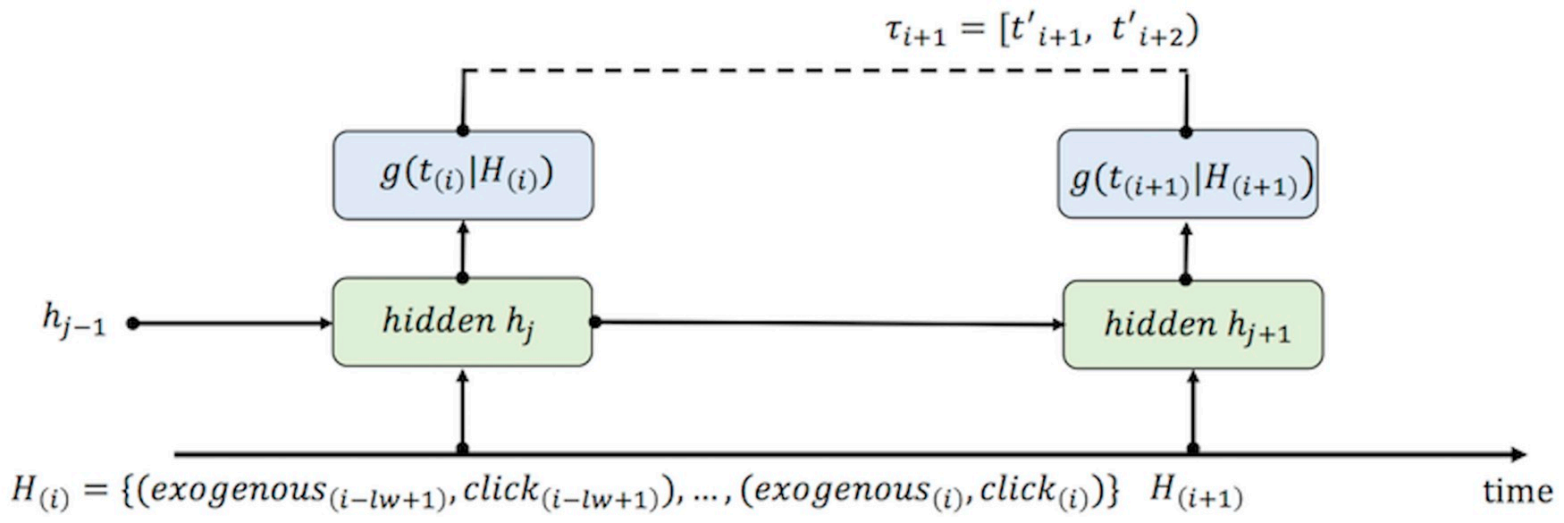
LSTM architecture. LSTM is good fit to both cases 1) where *eP*_*j*_ is equal to *t*_*i*_, (*i* = *j*), mathematical definition, and 2) where *eP*_*j*_ is equal to *t*_*i*_, (*i* ≠ *j*), technical implementation.

*LSTM* We derived the definition of Hawkes and the proposed kernel in a closed form in the previous section. By introducing required parameters qualified to sets, variables and functions for Hawkes in Table 1, we were able to set up the NLL of our generative model. Now, activity sequences defined in Fig (2) can be set as input data for a RNN to learn and forecast $T_{i}^{{}^{\prime }}$. We have chosen one layer LSTM and also notated required parameters as *lw*, lookback window, *H*_(*i*)_, history of activity sequences sampled *lw* in order, and *g*(*t*_(*i*)_|*H*_(*i*)_), function in the Neural Network. In the experiments with the real data sets, we configured conversion data as exogenous event. Fig 3 shows the architecture of LSTM configured in our method.

**Table 1:** Definitions.

Parameter	Interpretation
*α*	Increases intensity rate (*α* ≥ 1)
*δ*	Decreases intensity rate (*δ* ≥ 0)
*eP*_*j*_	Evaluation Point, as argument of λ(*t*)
*eP*	Set of *eP*_*j*_, equal to domain *t* *for* λ(*t*)
*J*	The index of the last element of the set *eP*
*t*_*i*_	Observed event time
*T*_*i*_	Set of *t*_*i*_ up to *i*
*I*	The index of the last element of the set *T*
$eP_{j}^{{}^{\prime }}$	Evaluation Point, as argument of λ(*t*) for the predictive period
*eP*′	Set of *eP*_*j*_, equal to domain of *t*λ(*t*) for the predictive period
*j*′	The index of the last element of the set *eP*′
$t_{i}^{{}^{\prime }}$	Forecasted event time
$T_{i}^{{}^{\prime }}$	Set of $t_{i}^{{}^{\prime }}$ up to i
*I*′	The index of the last element of the set $T_{i}^{{}^{\prime }}$
*τ*_*n*_	Distance [*t*_*i*_, *eP*_*j*_) such that *t*_*i*_ ≤ *eP*_*j*_
*γ*	Differential coefficient earned by gradient descent
	using Mean Squared Error (MSE) as objective function
*μ*(*t*)	Background intensity based on the observed activities,
	sets of view and click, during the configured look-back window at *t*
*θ*	Set of hyper parameters
*L*(*θ*)	Likelihood with parameters *θ*
*l*(*θ*)	Log likelihood function with parameters *θ*
*CIF*	Cumulative intensity function

#### LSTM-Hawkes model

LSTM-Hawkes is made up of four consecutive steps, A) model configuration, B) parameter optimization by minimizing Eq (12), C) earning differential coefficient *γ* which minimizes residual error, and D) forecasting posterior event time during the prediction period and drawing λ(*t*). Those steps and each algorithm are described in Algorithm 1, 2, 3, and 4 as pseudo codes below. In the Algorithm 1, we chose evaluation points by dividing the distance [*t*_1_, *t*_*n*_) with the size of *eP* which means at the implementation level, Δ*t* which is *t* − *t*_*i*_ in the Eq (8), is equal to *τ*_*n*_. After that, the hyper parameters are defined with the initial values and optimization is proceeded. In the Algorithm 4, by calculating lambda intensity with the results from LSTM prediction $T_{i}^{{}^{\prime }}$, our model finally draws cumulative intensity values for $\left[t_{1},t_{n}^{{}^{\prime }}\right)$.

**Algorithm 1: Model Configuration**

**Data:** Observed data set *t*_*i*_

**Result:** λ(*eP*_*j*_)

 *d* ← (*t*_*n*_ − *t*1)/*K*

 ⊳ *Set* *an* *equal* *interval*. *K* *is* *size* *of* *set* *eP*

 *eP*_1_ ← *t*_1_

**while** (2 ≤ *j* ≤ *K*) **do**

 *eP*_*j*_ ← *t*_1_ + (*j* − 1)*d*

 *j* = +1

 *α*, *δ* ← *α*_1_, *δ*_1_

 ⊳ *Hyper* *parameter* *setup*

**Algorithm 2: Parameter Optimization**

**Date:** λ(*eP*_*j*_)

**Result:** *α*, *δ*, *γ*

 *l*(*θ*) ← Eq (13)

 ⊳ *Set* *log* *likelihood*. *l*(*θ*)

**while** (*Not* *converged*) **do**

 *Run* *optimization* *function* *minimizing* −*l*(*θ*)

*Return Parameters of gradient exponential kernel α*, *δ*

**Algorithm 3: Earning Differential Coefficient *γ***

**Date:** Observed data *t*_*i*_

**Result:** *γ*

**for** *j* = 1; *j* ≤ *J*; *j* + + **do**

 **for** *i* = 1; *t*_*i*_ ≤ *eP*_*j*_; *i* + + **do**

  *CIF*.*Append*(λ(*ep*_*j*_ − *t*_*i*_))

*CIF* ← *Sum* *of* *CIF* *values* *by* *minute*

*OriginalIntensity* ← *Sum* *of* *observed* *event* *number* *by* *minute*

*n* ← *size* *of* *CIF*

**while** (1 ≤ *j* ≤ *n*) **do**

 Residuals = *Original*_*I*_ *ntensity*_*i*_ − *CIF*_*i*_

 *Optimization* *of* *Gradient* *Descent* *using* *MSE* *as* *loss* *function*

 *Return* *γ*

**Algorithm 4: Forecasting posterior event time** $t_{i}^{{}^{\prime }},\;\lambda (t_{i}^{{}^{\prime }})$

**Date:** *CIF* *of* *gradient* *hawkes*

**Result:** *CIF* *for* $\left[t_{1},\;t_{n}^{{}^{\prime }}\right)$

  *T*′ *LSTM Sampling*(*T, predictionPeriod*)

  $\;\;d\leftarrow \left(t_{n}^{{}^{\prime }}-t_{1}^{{}^{\prime }}\right)/K^{{}^{\prime }}$

  ⊳ *Set an equal interval*. *K*′ *is size of set eP*′

  $\;\;eP_{1}^{{}^{\prime }}\leftarrow t_{1}^{{}^{\prime }}$

**while** (2 ≤ *j* ≤ *K*′) **do**

  $eP_{j}^{{}^{\prime }}\leftarrow t_{1}^{{}^{\prime }}+\left(j-1\right)d$

  *j* = +1

**for** *j* = 1; *j* ≤ *J*′ *j* ++ **do**

 **for** *i* = 1; $t_{i}^{{}^{\prime }}\leq eP_{j}^{{}^{\prime }};\;i++$ **do**

  $CIF.Append(\lambda \left(ep_{j}^{{}^{\prime }}-t_{i}^{{}^{\prime }}\right))$

  *Return* *CIF*

  ⊳*CIF for* $\left[t_{1},\;t_{n}^{{}^{\prime }}\right)$

## Results and analysis

### Data

Criteo, an Ad Tech company, possesses cutting edge user re-targeting technology and has led the development of prediction methods based on machine learning algorithms. Being a leader in this field, Criteo has also notably hosted the Kaggle’s CTR forecast competition in 2014 and 2015. We applied the click and conversion data sets [29] provided by the Criteo Lab and compared the results between the the model/algorithm proposed and the Hawkes Intensity Process algorithm (HIP) [21] algorithm. We evaluated the forecasting accuracy of the posterior distribution of a click event from a single advertising network.

### Goodness of fit

**Fig 4:**
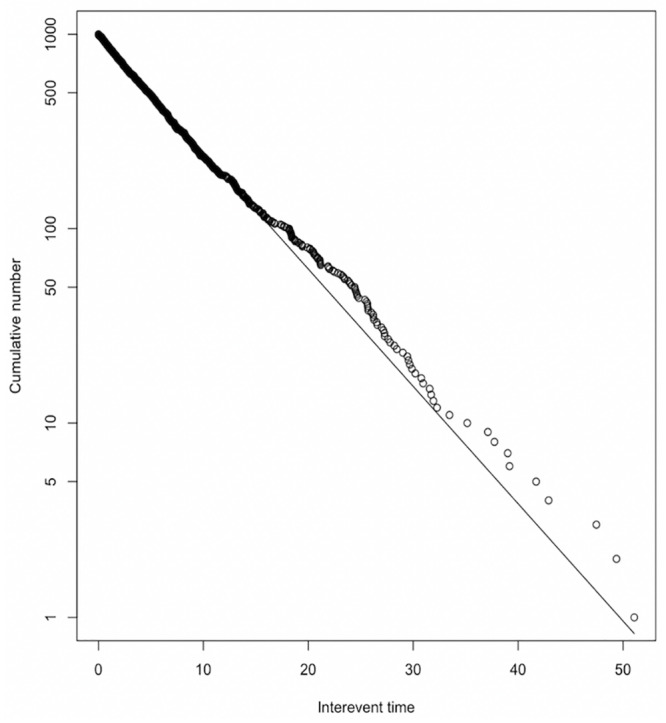
log-log-plot of residual’s interevent times. A thousand events are sampled.

Residual analysis [30] is a reliable measurement for the Hawkes model and has been widely used to evaluate the precision of a fit. Let *t*_*i*_ be a point process with intensity λ(*t*_*i*_) whose PDF is Eq (10) and *s*_*i*_ be equal to λ(*t*_*i*_) of Eq (5), then *s*_*i*_ becomes a unit rate Poisson process transformed by a Hawkes model. Thus, if the model fits well, the transformed process should resemble a unit Poisson process. Also, the residual’s inter-event time is supposed to be an independent exponential variable. Therefore, log-log-plot of the residual’s inter-event time should be close to the linear line. Fig 4 shows the log-log-plot of the residual inter-event time from the LSTM-Hawkes model and it proves that the shape of the distribution is very close to the linear line. Also, the first quintile of residual’s inter-event times are distributed the most. Thus, we can conclude that the LSTM-Hawkes model is an excellent way to determine a precise fit.

### Compared algorithm HIP

The HIP model is a Hawkes-based model that uses Power-Law-Kernel Eq (9). Since HIP mathematically induces the expectation function *ξ*(*t*) of λ(*t*) referring to exogenous events to predict endogenous event, it is great to compare with our model having the same input sequence. The only difference between two models is that HIP supposes that exogenous events are given even for the predictive period while LSTM-Hawkes does not.

$$\begin{array}{c} \xi \left(t\right)=Expectation\;of\;\lambda \left(t\right)\;where\;\lambda \left(t\right)=\mu s\left(t\right)+\sum_{t_{i}<t}km^{\beta }{\left(\tau +c\right)}^{-(l+\theta )}. \end{array}$$ (14)

### Compared algorithm Multivariate Linear Regression (MLR)

MLR has been widely adopted as an compared algorithm in population prediction in social media [31]. With production data we used for the experiment, next predicted intensity value based on the history *H*_(_ *i*) is calculated as below.

$$\begin{array}{c} f\left(H_{(}i\right))=\beta_{0}+exogenous_{i}*\beta_{1}+endogenous_{i}*\beta_{2} \end{array}$$

### Error score

$$s=\{\begin{array}{cc} \sum_{i=1}^{n}e^{-(d/a_{1})}-1 & for\;d<0,\\ \sum_{i=1}^{n}e^{\left(d/a_{2}\right)}-1 & for\;d\geq 0. \end{array}$$ (15)

Commonly used error measurements such as the MSE, Mean Absolute Error (MAE), and the Root Mean Square Error (RMSE) are only designed to measure the raw residuals. However, different from these methods, scores metric [32] is devised to take the negative error into account, which reduces the error score when the residuals are less than a certain point. Not only does the score metric method discover how many residuals the model produces, but it is also able to discover how well the model fits and predicts. In the experiment, we set up this certain point using the standard deviation of the observed data during the time period for the prediction. In the 1st test section, both *a*_1_ and *a*_2_ are 4.057 and in the 2nd test section both *a*_1_ and *a*_2_ are 7.495. The final score is the sum of each function conditioned on the distance mark.

### Experimental results

**Fig 5:**
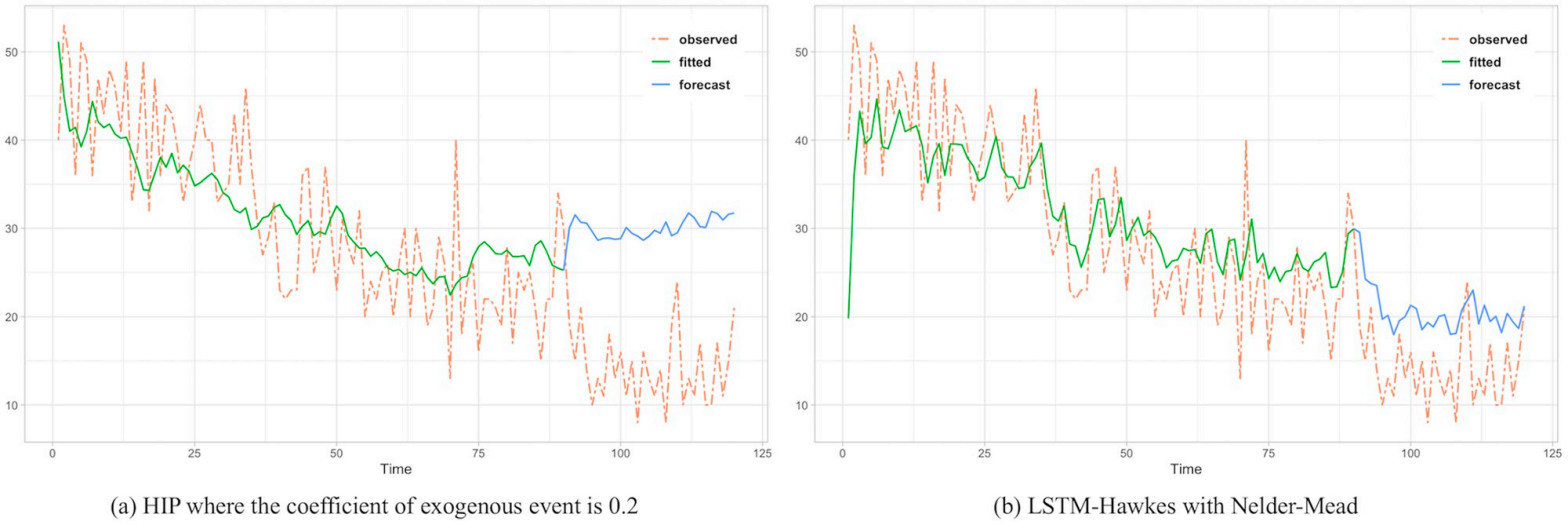
HIP with coefficient 0.2 shows the most MSE in HIP test in the 1st section as same as LSTM-Hawkes with SANN does in LSTM-Hawkes Test.

**Fig 6:**
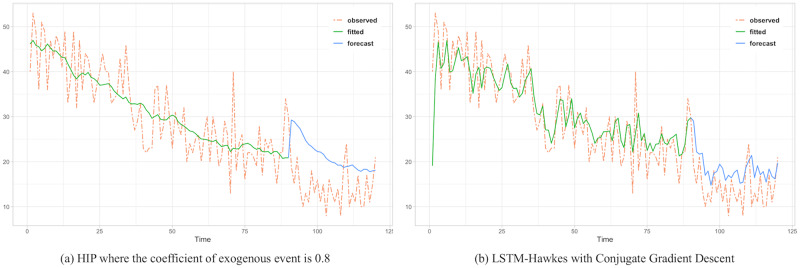
As correlation coefficient gets higher, HIP detects the sudden drop better. However since HIP *ξ*(*t*) is the expectation value of λ(*t*) with power-law decay, it tends to follow the trend a little more quadric rather than predict actual intensity value each time unit.

**Fig 7:**
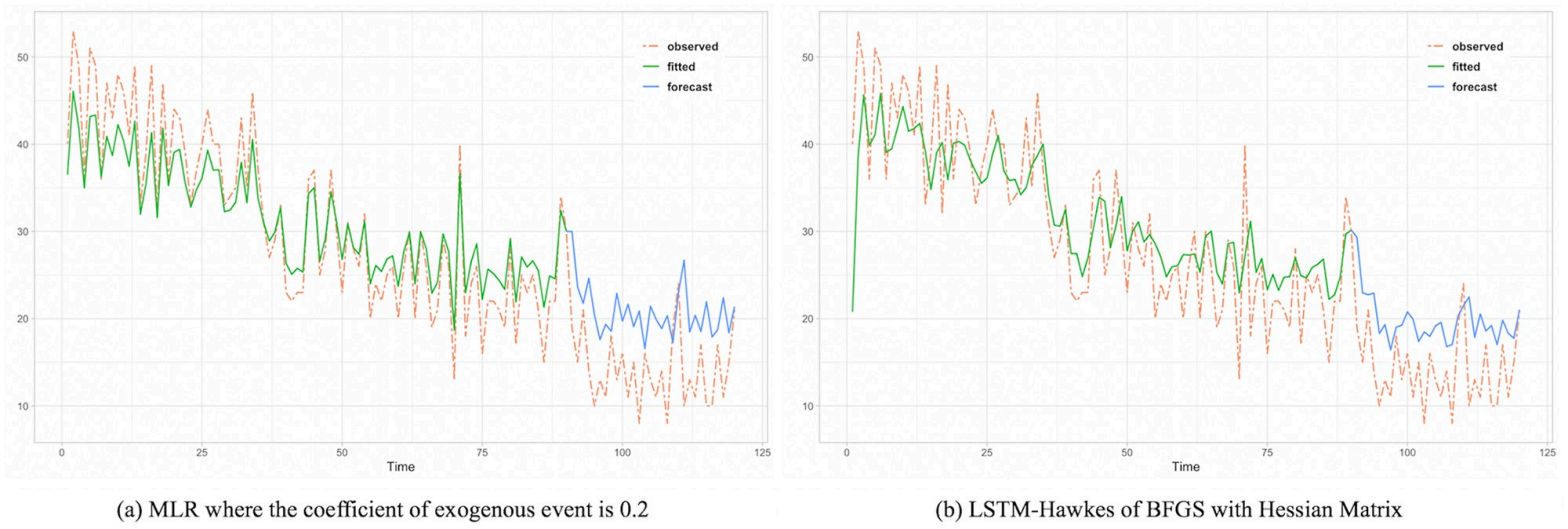
MLR shows overfitting issue and it still retains it in the period of prediction.

**Fig 8:**
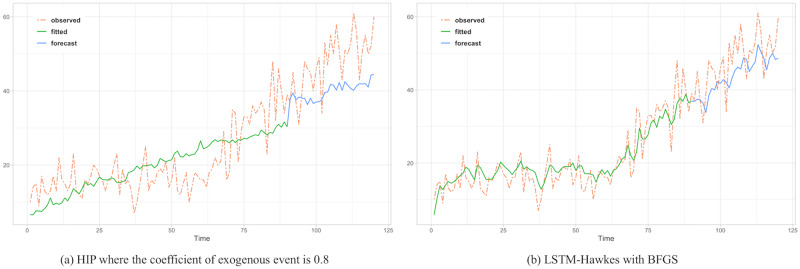
Rather than predictive period, it needs to compare the observed time period more. Since an under-fitted part is found with HIP result, prediction performance is quite noticeable.

**Fig 9:**
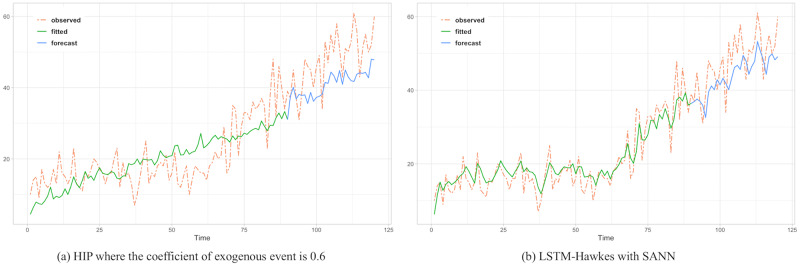
The under-fitted part is still found with HIP where the coefficient of exogenous event is 0.6 while both algorithm perform great in prediction resulting 76.9 and 40.7 MSE respectively.

**Fig 10:**
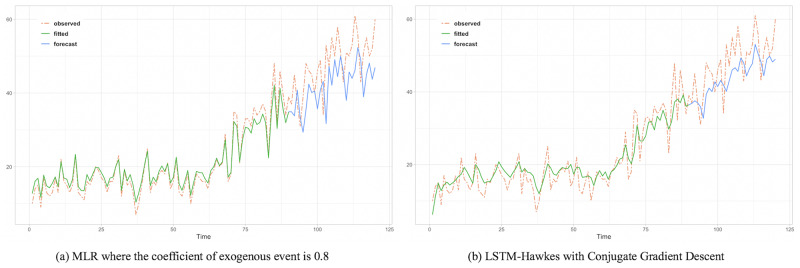
MLR shows over fitting problem in the learning period showing higher MSE.

To assess the comparison between the HIP, MLR and LSTM Hawkes models, we selected two different sections where the moving average trend of the click is monotonically decreasing in the first section and increasing in the second section. Test results of the first section are presented in Figs 5, 6 and 7 and Table 2. Also, test results of the second section are presented in Figs 8, 9 and 10 and Table 3. For an accurate performance assessment of the HIP model and MRL, we chose several correlation coefficients so that those models could show its accuracy in prediction, precision in fitting, as well as it’s limitation in both the fitting and prediction. For the accuracy of our assessment we chose four equally dispersed correlation coefficients of 0.2, 0.4, 0.6, and 0.8. The observed click is the endogenous event, and the exogenous event is the observed conversion.

**Table 2:** MSE and accuracy.

HIP		MLR	LSTM-Hawkes	
Correlation Coefficient	MSE	MSE	Method	MSE
0.20045197016606	272.86493629506	66.489085	Nelder-Mead	55.3646851328
0.40864585590557	167.08929263969	63.931454	BFGS with Hessian Matrix	31.8141777151
0.60923578698778	166.47562141766	62.859002	Conjugate Gradient Descent	44.5680031396
0.80069927612292	75.905022392	62.859002	SANN	47.1453170336
Avg Accuracy: 0.247342286175		Avg Accuracy: 0.4781933	Avg Accuracy: 0.531401427812	

MSE and Accuracy where the trend of moving average is monotonically decreasing.

**Table 3:** MSE and accuracy.

HIP		MLR	LSTM-Hawkes	
Correlation Coefficient	MSE	MSE	Method	MSE
0.20299594548747	801.44661263499	86.743375	Nelder-Mead	48.0034938190
0.40473704703628	101.32908279598	85.012468	BFGS with Hessian Matrix	41.3346206943
0.60048848461137	76.966689384563	84.804654	Conjugate Gradient Descent	42.6831118624
0.79396502231167	84.072450398227	84.804654	SANN	40.7298560311
Avg Accuracy: 0.741641169307		Avg Accuracy: 0.845652	Avg Accuracy: 0.882077335865	

MSE and Accuracy where the moving average trend is monotonically increasing.

First, in the forecasting test in the first section, it shows that the forecast made using the LSTM-Hawkes model reduces 73.9% of MSE on average and at least 27.1% compared to the HIP model where the correlation coefficient is 0.8, as well as 30.16% of MSE on average compared to the MLR. Fig 5(a) and 5(b) also shows the difference between the HIP and Hawkes models where a sudden drop occurs at the beginning part of prediction period. In contrast to HIP that does not follow the moving average due to the low correlation coefficient, LSTM-Hawkes follows the moving average closely, greatly reducing residuals. In Fig 6(a), HIP follows the moving average closely with a high coefficient (0.8) of exogenous data but still it’s MSE is at least 20 points higher than that of LSTM-Hawkes. MLR also shows great performance in fitting and learning. However this fitting capability eventually causes the overfitting issue and it is proved that LSTM-Hawkes reduces residuals more than MLR in Fig 10. The higher variance of observed data has, the more residuals the feature of MLR produces. Thus, in any case where the dynamics has bigger amplitude or higher frequency, measured MSE of MRL should be greater than the MSE of LSTM-Hawkes.

The forecasting test for the second section shows that LSTM-Hawkes reduces 83.8% of MSE on average and at least 37.5% compared to the HIP where the correlation coefficient is 0.6 as well as 49.39% of MSE on average compared to the MLR. Although both HIP (coefficient 0.6) and LSTM-Hawkes show great performance in prediction as can be seen in Fig 8, between 50 and 70 less-fitted parts are found with HIP while LSTM-Hawkes proves the goodness of fit. This gap is also shown in Fig 7(a) as well. From the tests, we were able to verify that our proposed model of the LSTM-Hawkes significantly outperforms the HIP model in both the fitting and forecasting criteria. As MLR showed the overfitting issue in the first experiment, at this time the MLR produces greater MSE again. Due to higher variance of the observed data of second section, MLR achieves an overfitted model with higher variance. While the compared algorithms show the similar performance in prediction (MLR’s fitting capability outperforms HIP), the LSTM-Hawkes outperforms the both compared methods in every experimental case.

**Fig 11:**
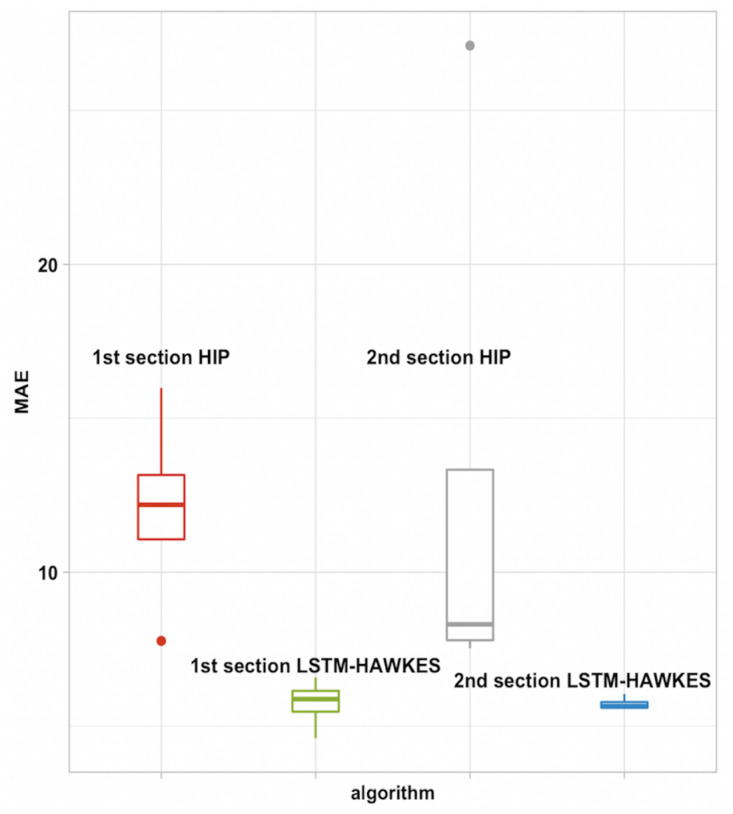
Comparability test. Full quantile range of Mean Absolute Error result of both HIP and LSTM-Hawkes.

Pertaining to the compatibility test with various maximum likelihood estimation algorithms based on both gradient descent method and Newtonian method, we could prove that LSTM-Hawkes has great compatibility with all of the tests. We set up our model for the compatibility test with Nelder-Mead [33], BFGS with Hessian Matrix [34], Conjugate Gradient Descent [35], and Simulated Annealing [36] and have our model optimized by estimating hyper parameters with the maximum likelihood estimation functions mentioned above. Fig 9 shows learning results based on these optimization functions and we could discover that the full interquartile range (IRQ) of MAE is a lot smaller than that of HIP (Fig 11). Also, since our model is not fully dependent with exogenous data to refer, the outlier-points cannot be found. It is expected that compatibility with different optimization algorithms will always be guaranteed.

**Fig 12:**
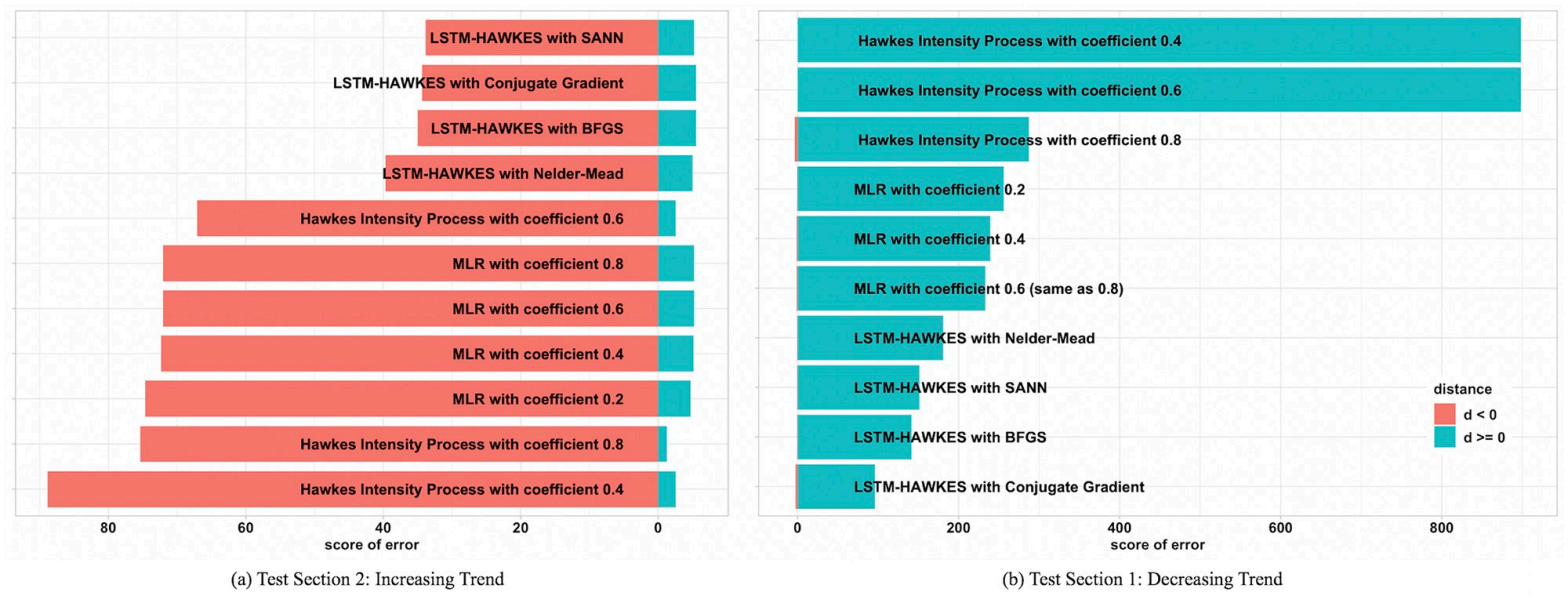
Under the circumstance that the moving average of ad clicks slopes downward, the HIP gets a higher error score where the distance mark *d* in Eq (15) is negative, and vice versa where the moving average of ad clicks slopes upward. HIP with the exogenous data with coefficient 0.2 is not included in the figure since it’s value is outlier, 2348.205812 in the downward trend and -1779.626 in the upward trend.

Lastly, when we assessed the score metric, Eq (15), of the two models, we were able to see that the LSTM-Hawkes model always obtained a greater percentage of negative error score than those of the HIP model and MLR presented in Fig 12. Thus, this always resulted in a lower score metric for the LSTM-Hawkes model, proving it’s greater accuracy of prediction and goodness of fit.

## Conclusion

### Conclusion

In the real production environment, an advertising network forecasting application does require the prediction results within milliseconds or even microseconds [37]. Therefore, the approach to the short interval prediction using Neural Network is not just scientifically important but also practically required since it does take great advantages in fast forecasting. Plus, because Algorithm (1), (2) are able to be performed in parallel or con-currently while Algorithm (3) is being conducted, the actual processing time in the production environment would expect to be even shorter. Thus, the LSTM-Hawkes using stochastic based generative model always guarantees the time efficiency.

### Discussion

These days, to tract more consumers, advertisers strongly focus on conversion events which actually create sales. Thus, the percentage of mobile advertising bidding based on conversion events increases. However, since conversion events rarely occurs, return Conversion Ratio (CVR) predicted based on a historical dataset is a great challenge for existing models including the Hawkes and other Neural Network models. However, different from the original mathematical definition of the kernel, we did not suppose that limitation of Δ*t* goes to zero. Therefore, we can easily expand the application of the model for the longer interval forecast based on bigger time unit. In future research, we plan to develop and implement a CVR prediction model, expanding to LSTM-Hawkes, which overcomes the data sparsity problem.

## Supporting information

S1 Dataset To help any of researchers to reproduce the result, we provide the codes of the model. Having the original data sets that can be found at http://labs.criteo.com/wp-content/uploads/2014/07/criteoconversionlogs.tar.gz, we first preprocess the data to conform behavior model (Fig 4) which can be found from the supporting information attachment. Then, we have processed Algorithm 1 to 4 in order to achieve the experimental results which also can be found from the supporting information attachment.
(ZIP)

S1 File (R)
