# Supplementary material for: A LSTM-Hawkes hybrid model for posterior click distribution forecast in the advertising network environment
Source: PLoS One. 2020 Jun 5;15(6):e0232887. doi: 10.1371/journal.pone.0232887 (PMC7274406; doi:10.1371/journal.pone.0232887)
Supplement: S1 Dataset — Having the original data sets that can be found at http://labs.criteo.com/wp-content/uploads/2014/07/criteoconversionlogs.tar.gz, we first preprocess the data to conform behavior model (Fig 4) which can be found from the supporting information attachment. Then, we have processed Algorithm 1 to 4 in order to achieve the experimental results which also can be found from the supporting information attachment. (ZIP) [file pone.0232887.s001.zip › gradientHawkes-master/gradientHawkes_lib.tgz!/gradientHawkes/html/00Index.html]

R: What the Package Does (One Line, Title Case)

# What the Package Does (One Line, Title Case)

---

## Documentation for package ‘gradientHawkes’ version 0.0.0.9000

- DESCRIPTION file.

## Help Pages

|  |  |
| --- | --- |
| add\_up | functions for the gradientHawkes model |
| aggregation | functions for the gradientHawkes model |
| cif | functions for the gradientHawkes model |
| forecast\_accuracy | functions for the gradientHawkes model |
| forecast\_MAE | functions for the gradientHawkes model |
| forecast\_MSE | functions for the gradientHawkes model |
| forecast\_score | functions for the gradientHawkes model |
| getConcatenated | functions for the gradientHawkes model |
| getDistance | functions for the gradientHawkes model |
| gradientHawkes | functions for the gradientHawkes model |
| gradientUpdate | functions for the gradientHawkes model |
| gradient\_gamma | functions for the gradientHawkes model |
| learning | functions for the gradientHawkes model |
| timeTransform | functions for the gradientHawkes model |
